# Instagram for audience engagement: an evaluation of CERC framework in the GCC nations for digital public health during the Covid-19 pandemic

**DOI:** 10.1186/s12889-024-18957-1

**Published:** 2024-06-13

**Authors:** Ghanem Ayed Elhersh, M. Laeeq Khan, Aqdas Malik, Maryam Al-Umairi, Haneen Khaled Alqawasmeh

**Affiliations:** 1 Department of Media and Communication, College of Liberal & Applied Arts, Stephen F. Austin State University, Nacogdoches, TX USA; 2 School of Media Arts & Studies, Scripps College of Communication, Ohio University, Athens, OH USA; 3 Department of Information Systems, Sultan Qaboos University, Muscat, Oman; 4 Department of Radio and Television, Yarmouk University, Irbid, Jordan

**Keywords:** CERC, Public health, Crisis, Social media, COVID-19, Instagram

## Abstract

**Background:**

In this study, we investigate the utilization of Instagram by public health ministries across the Gulf Cooperation Council (GCC) nations to disseminate health-related information during the COVID-19 pandemic. With Instagram’s visual-centric approach and high user engagement, the research aims to investigate its critical yet complex role in information dissemination amid a health crisis.

**Methods:**

To examine how Instagram communication strategies align with the CDC’s Crisis and Emergency Risk Communication (CERC) framework, we employ the content analysis method. This approach helps to evaluate the effectiveness and challenges of employing Instagram for health communication within a region known for its significant social media usage.

**Results:**

Findings indicate that Instagram serves as a vital platform for the rapid dissemination of health information in the GCC, leveraging its visual capabilities and wide reach. The GCC ministries of health utilized Instagram to demonstrate a consistent and strategic approach to communicate essential COVID-19 related information. Kuwait and Bahrain were the most active of all the assessed ministries with respect to the number of engagement metrics (likes and comments). Most of the posts, as per the CERC framework, were informational and related to vaccine infection and death cases. The second most salient theme in line with the CERC framework was about promoting actions, followed by Instagram posts about activities, events, and campaigns.

**Conclusions:**

The research underscores Instagram’s potential as a powerful tool in enhancing public health resilience and responsiveness during health emergencies in the GCC. It suggests that leveraging social media, with careful consideration of its affordances, can contribute significantly to effective health communication strategies in times of crisis.

## Introduction

The COVID-19 pandemic underscored the critical yet complex role of technology and social media in disseminating health-related information, revealing it as a double-edged sword where the line between information and misinformation blurs easily. This dynamic emphasizes the undeniable influence of social media as a key vector for information dissemination, highlighted by recent statistics from January 2024, which indicate that a staggering 66.2% of the global population, equating to 5.35 billion individuals, are internet users, with 5.04 billion of them actively engaging on social media platforms [1]. The capacity of these platforms to facilitate the creation, sharing, and interaction with content has proven to be superior to traditional means of information dissemination, especially in the context of health-related content, where rapid sharing of information, suggestions, and issue reporting is crucial [2, 3].

However, this efficient dissemination mechanism also introduces significant risks of misinformation, which can mislead the public and exacerbate health crises. Some research revealed that social media significantly influenced public health behavior during the COVID-19 pandemic, with over 60% of participants reporting changes in their health practices based on information obtained from these platforms [4]. This is juxtaposed with findings from a KFF survey, which reports widespread exposure to health misinformation, highlighting a “muddled middle” segment of the population that is particularly vulnerable to misinformation [5].

The World Health Organization (WHO) has termed this phenomenon of rampant misinformation as an ‘infodemic,’ posing a substantial challenge to public health agencies in the digital age. While extensive research has explored the role of social media in health information dissemination within Western contexts [6–9], there remains a conspicuous gap in our understanding of its impact and application across diverse socio-cultural landscapes, particularly in the Gulf Cooperation Council (GCC) region. This gap is significant given the high social media penetration rates in the GCC and the crucial role these platforms play in disseminating information and shaping public health strategies during crises.

Therefore, this research addresses the current knowledge gap by investigating how GCC countries utilize Instagram to communicate health-related information to the public during the pandemic. Instagram, a visual-centric social media platform, experienced significant growth, with visuals proven to be more memorable and engaging. Research underpins the impact of Instagram’s visual and interactive attributes on public health communication strategies, showing that image-based posts generally attract more engagement, thus broadening the reach of vital health advisories [10–12].

The utilization of social media for public health communication in the GCC region mirrors global trends in many ways, yet also displays distinct regional characteristics that reflect its unique socio-cultural landscape. For instance, during the COVID-19 pandemic, global trends showed a rapid increase in the use of social media platforms by health organizations to disseminate information swiftly and engage with the public directly.

The GCC countries boast some of the highest social media usage rates globally, with Instagram being one of the most popular platforms with over 30 million users. Specifically, the platform has 16.30 million users in the Kingdom of Saudi Arabia (KSA), above 7.0 million users in the UAE, 2.80 million in Kuwait, 2.40 million in Oman, 1.65 million in Qatar, and 1.1 million in Bahrain. This extensive reach provides an unprecedented opportunity for public health agencies to communicate directly with a significant portion of the population, making it a critical medium for health communication efforts [7]. Also, Instagram allows for the culturally appropriate tailoring of public health messages, which can increase message acceptance and engagement.

Furthermore, the GCC’s high penetration rates of social media facilitated an unprecedented reach of health advisories, which were often delivered in both Arabic and English to cater to the diverse expatriate population, unlike the predominantly monolingual approaches observed in many Western regions. This nuanced adaptation of global trends to local preferences underscores the strategic significance of understanding regional differences in social media use, which this study seeks to explore further.

This study utilizes content analysis of Instagram content to examine how GCC Instagram strategies align with the Crisis and Emergency Risk Communication (CERC) framework developed by the United States Centers for Disease Control and Prevention (CDC). The CERC framework is specifically designed for effective communication during crises and is structured around five key stages: 1) Pre-crisis, where preparedness and prevention are emphasized; 2) Initial, focusing on immediate response and public alert; 3) Maintenance, to sustain communication and public trust throughout the event; 4) Resolution, as the crisis begins to abate; and 5) Evaluation, assessing the response to improve future communication efforts [13]. This structured approach aids in managing public communication during health emergencies, and this research aims to assess its implementation in digital communications strategies.

Investigating how Instagram is used for public health messaging in the GCC highlights its strategic significance and the effects it has had during pandemics. This not only allows for a wider comparison with other regions but also helps improve our global understanding of the challenges and successes of digital health communication. By delving into these dynamics, we can develop more effective digital strategies and policies tailored to public health emergencies. Ultimately, this research could lead to practical, evidence-based advice on using social media to bolster public health efforts and responses.

## Literature review

### Social media and public health

In today’s digital world, social media has emerged as a transformative force, reshaping how information is disseminated and consumed. Its impact is particularly palpable in the realm of public health, where it serves as both a catalyst for promoting health awareness and a conduit for the rapid spread of information [14]. Social media’s dual capacity to empower and challenge public health initiatives underscores its complex role in contemporary health communication.

Platforms like Instagram, X (formerly Twitter), and Facebook have proven vital tools in promoting public health awareness [15–17]. They break down geographical and socio-economic barriers, offering unprecedented opportunities to convey important health messages to a global audience [18, 19]. For instance, the COVID-19 pandemic highlighted social media’s significance, with these platforms facilitating the widespread dissemination of critical information about the virus, preventive measures, and vaccination campaigns [20]. Through live sessions with health experts, interactive Q&A segments, and targeted hashtags, social media has played a crucial role in educating the public [21].

The rise of health influencers and bloggers on social media has introduced a new dimension to health communication. These individuals have significantly influenced health behaviors and attitudes through their posts on fitness, nutrition, and mental health self-care [22]. However, the varying credibility of these influencers and the accuracy of their information necessitate a critical evaluation by their audience. Some researchers have discussed that campaigns like #BellLetsTalk in Canada exemplify social media’s positive impact on public health awareness [23]. By leveraging social media’s viral nature, such campaigns have successfully fostered open conversations about mental health and encouraged individuals to share their experiences, thereby creating a supportive community [23]. Furthermore, social media data analytics and monitoring systems have given researchers and policymakers real-time insights into public health trends and community health statuses [24, 25].

Social media has also become a dynamic tool for traditional health institutions aiming to achieve various health goals. It offers innovative ways to promote healthy habits and facilitates a more efficient and participatory approach to health communication [19]. Health organizations have embraced social media for its ability to enhance process documentation, increase individual participation, and improve overall efficiency. It has also revolutionized the way people seek, discuss, and share health information. Its widespread use has made health information more accessible, helping to solve health problems at minimal cost and significantly increasing general health awareness [26].

As social media continues to evolve, its applications in the health sector are bound to expand, offering significant benefits for public health communication, policy-making, and community engagement [27]. This forward-thinking integration of social media into health strategies promises to address public health challenges effectively, leveraging digital innovation to foster a healthier, more informed society [27]. However, despite these substantial benefits, the relationship between social media and public health awareness is fraught with challenges, most notably the spread of misinformation. This complex situation underscores the critical importance of relying on verified and reliable sources for health-related information, ensuring that the public receives accurate guidance and support in navigating health decisions [27, 28].

### Social media’s role in crisis communication

The emergence of social media since the late 1990s has been a game-changer in various sectors, including crisis and risk management. Its capacity to merge diverse societal segments has not only democratized information dissemination but also transformed emergency response strategies [29]. Social media’s unique attributes—immediacy, accessibility, and interactivity—have significantly bolstered the effectiveness of communication between the public and official agencies during crises [30, 31].

In recent years, platforms like Instagram have become essential in disseminating information during emergencies, from natural disasters like the 2010 Haiti earthquake [32] to global health crises such as the COVID-19 pandemic [7], and social media content and engagement strategies [33]. The visual-centric nature of Instagram, combined with its vast user base, facilitates rapid information sharing and public mobilization in times of need [10, 11]. However, the boon of social media comes with a caveat: the challenge of misinformation. As quickly as accurate information spreads, so too can falsehoods, making it crucial for authorities to provide timely and trustworthy updates [34, 35].

The COVID-19 pandemic underscored the indispensable role of social media in crisis communication. Health organizations, including the WHO and CDC, harnessed platforms like Instagram for timely updates, combating rumors and myths while fostering a sense of community resilience. This approach exemplified an efficient use of social media, balancing the rapid dissemination of information with the need for accuracy and credibility [7, 36].

Social media has not only provided a platform for official updates but also facilitated peer-to-peer communication, enabling the public to share experiences, support, and advice. This dual function enhances public engagement and awareness, contributing to a more informed society capable of responding effectively to emergencies [37, 38]. The integration of social media into crisis management frameworks reflects an acknowledgment of its critical role in modern communication strategies. The Crisis and Emergency Risk Communication (CERC) framework emphasizes principles such as being first, accurate, and credible—ideals that social media can embody when used effectively. Applying these principles, health organizations have demonstrated social media’s potential to guide public behavior and perceptions through empathetic and action-oriented communication [39].

As social media continues to evolve, so too will its role in crisis communication. The challenge for public health agencies and crisis managers lies not only in leveraging social media’s expansive reach but also in navigating the complexities of misinformation. Ensuring that social media serves as a force for good in crisis communication will require ongoing evaluation, innovation, strategic engagement, and a commitment to accuracy and transparency. In the contemporary era of digital technology, the ability to communicate effectively during emergencies is more critical than ever, highlighting the need for a nuanced understanding of social media’s power and pitfalls.

### COVID-19: the case of GCC

**Fig. 1:**
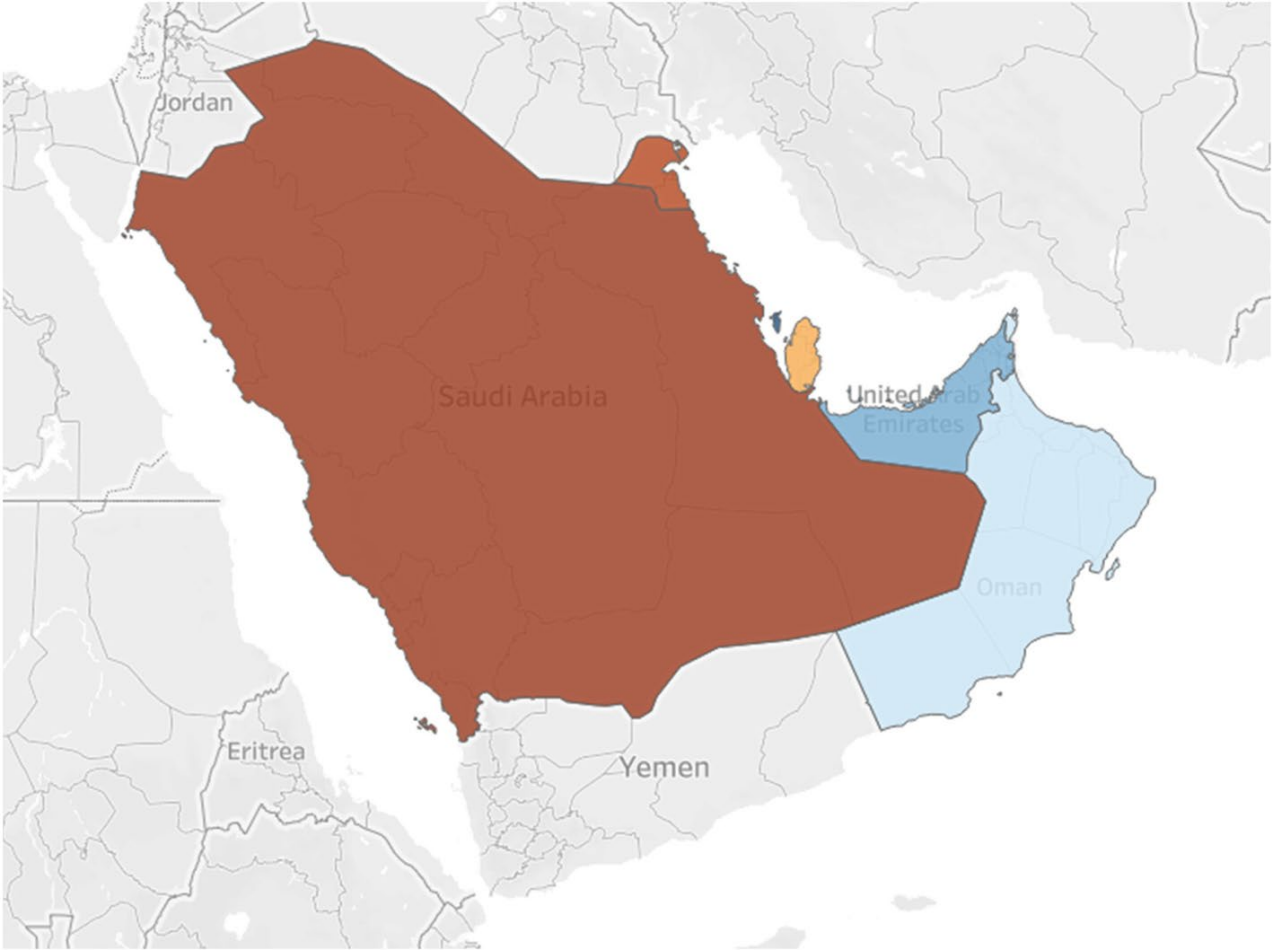
GCC map (Creative Commons License)

The Gulf Cooperation Council (GCC), established in Riyadh, Kingdom of Saudi Arabia (KSA) in May 1981, is a regional political and economic union encompassing six Arab states of the Persian Gulf: Bahrain, Kuwait, Oman, Qatar, KSA, and the United Arab Emirates [40] as stated in Fig. 1. These countries are united by their common goals, cultural and political identities deeply rooted in Arab and Islamic traditions. With capitals ranging from Manama in Bahrain to Abu Dhabi in the UAE, the GCC plays a significant role in the region’s stability, economic development, and cultural exchange, housing a combined population of over 55 million people as of early 2023 [40].

Social media usage in the Gulf Cooperation Council (GCC) countries is marked by impressive figures, with the United Arab Emirates leading the charge at a 99% penetration rate among internet users, closely followed by Saudi Arabia at 72%. These statistics reflect a broader trend across the region, where countries like Qatar, Kuwait, Oman, and Bahrain also show substantial engagement on various platforms. The diversity in platform preferences, from Instagram and LinkedIn being favorites in the UAE to Snapchat and X (formerly Twitter) being popular in Saudi Arabia, underscores the GCC’s significant and varied digital landscape. This rapid adoption of social media across the GCC is further highlighted by the 2023 report from Global Media Insight, evidencing the region's deepening digital connectivity [41].

As the coronavirus pandemic unfolded in late 2019, it plunged the global community into a rapidly escalating health crisis, necessitating unprecedented measures including travel bans, mandatory home isolation, and quarantine for those potentially exposed to the virus. The GCC countries, in line with global trends, faced significant outbreaks, marking them as areas with notably high infection rates relative to their populations during the pandemic. The number of infected cases in various (GCC) countries during the second wave until December 2021 was high. For instance, Saudi Arabia (KSA) has the highest reported cases at 362,488, indicating a significant impact of the health issue within its borders. The United Arab Emirates (UAE) follows with 204,369 cases, suggesting a relatively high number of cases as well. The data for Oman shows 128,633 cases, whereas Qatar has slightly more cases at 143,428. Bahrain and Kuwait report lower figures than their neighbors, with Bahrain having 92,169 cases and Kuwait having 150,093 cases.

The COVID-19 pandemic marked a significant shift in how the world communicates health information, with the internet becoming a primary resource. Before the pandemic, research showed that a large part of our daily interactions were online, with three out of four internet searches related to health information [42, 43]. This trend intensified after the pandemic began, as people turned to the web for details on symptoms and health practices. Approximately 10.2 million global searches for “COVID,” highlighting the surge in online health information seeking [44].

Digital platforms, particularly social media, played a pivotal role in public health communication during the COVID-19 crisis. The WHO leveraged social media to disseminate science-based content, engage with the public, and combat misinformation [36, 45]. This approach was echoed by health institutions globally, recognizing social media as a critical tool for crisis communication, enabling timely and effective public awareness campaigns [46]. The American Red Cross president emphasized the transformative impact of the social web on disaster response, with usage spiking by 61% during lockdowns [47].

Following the actions of WHO and CDC, the Gulf Cooperation Council (GCC) countries used social media platforms, particularly Instagram, to communicate COVID-19-related information to the public. The region saw a significant increase in Instagram users, with over 30 million accounts by early 2024, reflecting its popularity among a young demographic [48]. This trend underscores the critical need to examine the GCC Ministries of Health’s (MOHs) use of Instagram for conveying COVID-19 prevention and updates, considering its visual communication strengths and widespread reach [7].

### Theoretical framework: CERC

The Crisis Emergencies and Risk Communication (CERC) Framework represents a pivotal approach to managing communications during crises and risk-laden situations. Developed to guide health organizations through the complexities of disseminating critical information amidst uncertainty, CERC has proven instrumental in uncovering the unknown by leveraging available knowledge [39]. Distinctively, the framework demystifies risk-related incidents, categorizing them into specific stages to facilitate clear and concise public communication [49]. While alternative frameworks exist, they typically lack the nuanced consideration of a crisis's evolving stages, offering only broad directives for health authorities’ actions [3].

**Fig. 2:**
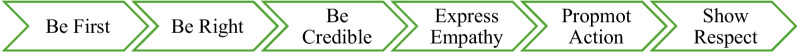
CERC principles

Internationally recognized and disseminated by the CDC, CERC stands on a foundation of empirical research. It articulates six core principles essential for effective crisis communication, as depicted in Fig. 2. These principles are encapsulated in the framework’s comprehensive model [50].

**Fig. 3:**
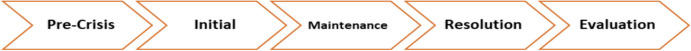
CERC stages

The (CERC) core principles, when combined, create a comprehensive framework for efficient public communication during disasters. This framework ensures the rapid, precise, and compassionate broadcast of information while also promoting public empowerment and mutual respect. Furthermore, the framework is unique in how well it handles crises because it is based on strong theories, takes a comprehensive view, has been improved through empirical research and practical experiments, and carefully takes into account the different stages of a crisis [39]. It methodically divides crises into five distinct stages—(1) Pre-Crisis, (2) Initial, (3) Maintenance, (4) Resolution, and (5) Evaluation. Each stage is paired with a strategic plan designed to guide organizations in crafting and executing responses that are both appropriate and efficacious [13], as depicted in Fig. 3. This structured approach not only facilitates a systematic response but also aids in the effective management and resolution of crises.

Each stage within the Crisis Emergencies and Risk Communication (CERC) framework dictates a distinct communication strategy tailored to the evolving circumstances of a crisis. During the *Pre-Crisis phase*, the primary objective is to inform the public about potential threats, offering guidance on preparatory actions should those threats escalate into a crisis [13]. The crucial *Initial phase* signifies the onset of the crisis [13], focusing on reducing uncertainty, enhancing reassurance, and fostering self-efficacy among the public. In the *Maintenance phase*, efforts shift towards correcting misinformation and providing the public with detailed updates on the crisis’s progression. This continuous flow of accurate information is vital for maintaining trust and managing public perception. As the crisis transitions into the *Resolution phase*, the focus of communication moves towards recovery, detailing rehabilitation efforts and restoration initiatives and elucidating the crisis’s root causes [7, 13]. Finally, the *Evaluation phase* offers a moment for reflection and learning, where authorities assess the crisis management efforts, identifying key lessons and evaluating the efficacy of the communication strategies employed throughout the crisis lifecycle [13]. It is important to note that while the framework stages are laid out sequentially, real-world applications may see phases overlapping or recurring, influenced by a nation's specific environmental factors or practices [39].

In a comprehensive study led by Lundgren and Andrea, the CERC framework was meticulously examined, identifying six distinct classes for organizing the dissemination of information to the public by authorities during health crises [51]. These classifications—*Risk Messages, Warnings, Preparation, Uncertainty Reduction, Efficacy, and Reassurance*—have been fundamental in guiding numerous subsequent studies on the CERC approach, thereby reinforcing its pivotal role in health communication. By detailing crisis signs, exacerbating factors, first responder actions, safety recommendations, and emphasizing personal safety and communal responsibility, the framework aims to mitigate health risks and encourage a shift in public behavior towards increased preparedness and resilience. Echoing this sentiment, [52] highlighted the framework’s ability not only to prevent health issues from worsening but also to foster a sense of self-efficacy and collective empowerment among individuals. Through effective communication, the CERC framework aims to convince the public of their capacity to face and surmount challenges, thus playing an essential role in improving public safety and health outcomes during crises. This approach underscores the importance of clear, strategic communication in creating an atmosphere of informed readiness and proactive community engagement.

After reviewing current studies within the same domain, Table 1 showcases key research evaluating health institutions’ communications during times of crisis. The studies compiled in Table 1, demonstrate diverse themes, from risk understanding to self-efficacy and sense-making, and integrate preparation with uncertainty reduction to tackle misinformation, governmental actions, and expressions of gratitude, while advisories highlight warnings and guidance. Despite using qualitative content analysis and focusing on global health entities like the WHO and the CDC, variations arise from the specific objectives and timelines of each study, affecting the data collected. The findings emphasize social media’s pivotal role in disseminating health messages during crises, revealing both adherence and gaps in applying the CERC model. This sets the stage for further research into the use of Instagram by GCC MOHs within the CERC framework, aiming to enhance qualitative research methodologies and communication effectiveness.

**Table 1:** Salient research studies within the domain

Author, Year, Title	Platform & data	Country	Key findings
(Mohamed, 2021) An Analysis Of Risk Communication By The Finnish And Scottish Government On Twitter During The Covid-19 Pandemic	Twitter, 330 Scottish tweets & 146 Finnish tweets	Scotland &Finland	1- Social media is a useful tool for risk communication 2- from a CERC perspective, gaps in risk communication strategies were identified 3- both governments hardly used their Twitter platforms to address misinformation or rumors
(Malik et al., 2021) Public health agencies outreach through Instagram during the COVID-19 pandemic: Crisis and Emergency Risk Communication perspective	Instagram, 296 posts	International	1- Instagram can be an effective communication tool to convey health messages during a crisis 2- Most of the analyzed posts were representing celebrities, clarifications, and infographics 3- By providing accurate information and credible sources, social media can create strengthen opportunities to counter health misinformation
(ElGohary, 2023) Tweeting During Emergencies: The Egyptian Ministry of Health Twitter Communication Strategy Under the Umbrella of the Crisis and Emergency Risk Communication Model (CERC) CASE STUDY: COVID-19 Pandemic	1825 tweets	Egypt	With different degrees of frequency, the Egyptian Ministry of Health was successful in implementing most, if not all, of the advised message and communication characteristics; nevertheless, it did not adhere to the advised application order of stages
(Nagahawatta et al., 2022) Strategic Use of Social Media in COVID-19 Pandemic. Pandemic Management by Sri Lankan Leaders and Health Organizations Using the CERC Model	111 Facebook posts and 64 LinkedIn posts	Sri Lanka	The adaptability of the Crisis and Emergency Risk Communication Model (CERC) to improve communication during a crisis by leaders and health-focused organizations
(Alhassan & AlDossary, 2021) The Saudi Ministry of Health’s Twitter Communication Strategies and Public Engagement During the COVID-19 Pandemic: Content Analysis Study	Twitter, 1217 tweets	Saudi Arabia	While the usage of hashtags was related to a higher level of public involvement, the inclusion of hyperlinks and multimedia files had a detrimental impact on engagement. Public interaction was high when warnings, reducing uncertainty, and reassuring tweets were posted
(Guidry et al., 2017) Ebola on Instagram and Twitter: How health organizations address the health crisis in their social media engagement	Twitter (779 Tweets) & Instagram (107 posts)	International	Instagram can be a useful platform for establishing meaningful, interactive communication with the public in times of global health crises, as evidenced by significantly greater levels of engagement on the part of health organizations and the public
(MacKay et al., 2021) Examining Social Media Crisis Communication during Early COVID-19 from Public Health and News Media for Quality, Content, and Corresponding Public Sentiment	Facebook, 438 posts	Canada	1- Form Five guiding principles of effective crisis communication And public trust using social media best practices 2- Presents three Key features of crisis communication topics

The COVID-19 pandemic has served as a watershed moment for public health communication across the globe, underscoring the critical role of digital platforms in disseminating vital health information. Following the actions of WHO and CDC, the Gulf Cooperation Council (GCC) countries used social media platforms, particularly Instagram, to communicate COVID-19-related information to the public. This focus on Instagram for health communication reflects a broader trend in integrating social media into public health strategies [7]. Therefore, the importance of investigating the GCC Ministries of Health’s (MOHs) use of Instagram lies in its unique demographic engagement, visual communication capabilities, and widespread popularity in the region, making it an effective tool for reaching and educating the population on COVID-19 prevention and updates. Thus, this research seeks to answer the following questions:

**RQ1**: In what way do the ministries of health (MOHs) in the GCC nations utilize Instagram to communicate COVID-19-related messages?

**RQ2**: What is the nature of the content disseminated by the ministries of health (MOHs) in the GCC nations on Instagram, and to what extent does this content align with the principles of the Crisis and Emergency Risk Communication (CERC) framework?

**RQ3**: How does online audience engagement manifest itself on Instagram content in terms of likes and comments for the MOHs in the GCC nations?

## Methods

### Sample and data collection

In this study, we systematically explored the use of Instagram by Ministries of Health (MOHs) of the Gulf Cooperation Council (GCC) during the COVID-19 crisis. We adhered to the Crisis and Emergency Risk Communication (CERC) framework to identify the maintenance phase from official GCC MOH Instagram accounts (@Omanimoh, @Kuwait_Moh, @Saudimoh, @Mophqatar, @Mohbahrain, and @Mohapuae). See Table 2.

**Table 2:** MOHs on Instagram in the GCC

Country	Account	Date Of Joining Instagram	Date Of Verification	No. Of Posts * Till May 2023	No. Of Followers *Till May 2023
Sultanate Of Oman	@Omanimoh	Jul 2016	Feb 2021	5,915	163 k
Kuwait	@Kuwait_Moh	Dec 2012	Jun 2020	2,737	644 k
Kingdom Of Saudi Arabia	@Saudimoh	Sep 2015	Apr 2020	1,659	967 k
Qatar	@Mophqatar	Mar 2020	Mar 2020	2,799	177 k
Kingdom Of Bahrain	@Mohbahrain	Aug 2013	Jan 2020	13,039	269 k
United Arab Emirates	@Mohapuae	Aug 2013	Feb 2022	11,393	320 k

CrowdTangle, a social media tracking tool developed by Meta, was utilized for this purpose. This tool enables researchers to monitor, analyze, and report on content across public pages and groups on platforms like Instagram and Facebook, featuring a comprehensive search function that allows for an exhaustive examination of the public Facebook and Instagram dataset [53, 54]. By entering specific search terms and setting the search parameters, users can retrieve data spanning a defined period [53].

The search queries used for this study involved the terms “كوفيد-١٩” and “COVID-19,” spanning from May 1, 2021, to April 1, 2022—aligned with the peak of COVID-19 cases in the region—. After inputting these details into CrowdTangle, we identified a total of 7,223 posts. The collected data were organized using Microsoft SQL Management Studio, allowing us to focus on posts that resonated most with audiences in terms of COVID-19-related engagement.

Subsequently, for inclusion purposes, we eliminated posts using COVID-19 hashtags that did not pertain to the actual topic, and we also excluded videos to focus solely on still images. To fairly assess the efforts of GCC countries during this pandemic phase, we selected an equal number of posts from each account, specifically choosing those with the highest engagement in terms of likes and comments.

We then meticulously selected a total of 900 posts, 150 for each country, using purposive sampling to align with our objective of equally representing governmental communication efforts across all GCC countries. Table 3 below displays sample images from the official Instagram accounts of the GCC Ministries of Health. Each post was manually reviewed to identify and categorize dominant themes. These were then systematically documented in a separate Google forum for each country, facilitating a detailed analysis of how the Ministries of Health in the GCC utilized Instagram for public health communication during the pandemic.

**Table 3:** Sample images from Instagram posts of the GCC nations health ministries

Oman	Kuwait
Bahrain	KSA
Qatar	UAE

### Developing the content analysis code book

To address the first research question (RQ1), this study synthesized a comprehensive codebook by examining and integrating content analysis codebooks from various related studies. This newly developed codebook consolidates recurring themes into unified categories and assigns descriptive labels to each. It draws upon the existing body of literature, including the influential studies [7, 51, 55], which share a contextual similarity with this research. As illustrated in Appendix A, the codebook captures a wide range of themes, from symptom awareness to governmental measures and communal obligations, ensuring a tailored and thorough thematic categorization of the collected data.

The codebook, as outlined in Appendix A, aided in categorizing the information into distinct themes, including essential COVID-19 messages, promotion of actions, events, and campaigns, reassurance, collective responsibility, and a general category for content that does not fit into these themes. It provides further information by classifying the sort of visual content (either infographic or photograph), the identities of the featured individuals (celebrity, caregiver, ordinary individual, or undefined), and their gender (male, female, both, or none). In addition, a third dimension of analysis was utilized to document the number of likes on postings, facilitating an investigation into the relationship between different forms of material and the level of public involvement. This analysis specifically targeted the second research question (RQ2), focusing on categorizing the number of likes into three ranges: less than 1000, 1001–5000, and over 5001 likes.

A random sample of COVID-19-related and most liked posts from each country was analyzed to ensure the codebook’s inclusiveness and applicability across all collected content. This initial analysis provided a tentative assessment of the codebook’s coverage of necessary themes. It led to slight modifications, such as introducing a general category to include posts that did not align with other categories. The subsequent analysis focused on further classifying posts by their visual content, the presence and identity of individuals, and their engagement levels to address the study’s second question regarding the relationship between content and public engagement.

The codebook’s efficacy and reliability were confirmed by coding a set of 90 initial posts. This preliminary coding served as a critical foundation for the consistent application of the codebook throughout the research. The third and fourth authors, who focused primarily on the visual content of the posts, conducted the coding. Textual captions were used only as supplementary information when the imagery alone was insufficient for making coding decisions. The coders reached a consensus on the application of coding criteria through extensive and detailed discussions, resulting in a Cohen’s Kappa reliability score of over 0.7 for each category. This significant score demonstrates a high level of agreement and consistency between coders. The detailed process of achieving this consensus, including examples of discussions and adjustments to the codebook, underscored the robust validation of our coding methodology. As outlined by [56], this rigorous validation process was crucial for ensuring the precision and reliability of the coding, confirming that the coding categories were well-defined and that the final analysis was both accurate and trustworthy.

## Results

**Fig. 4:**
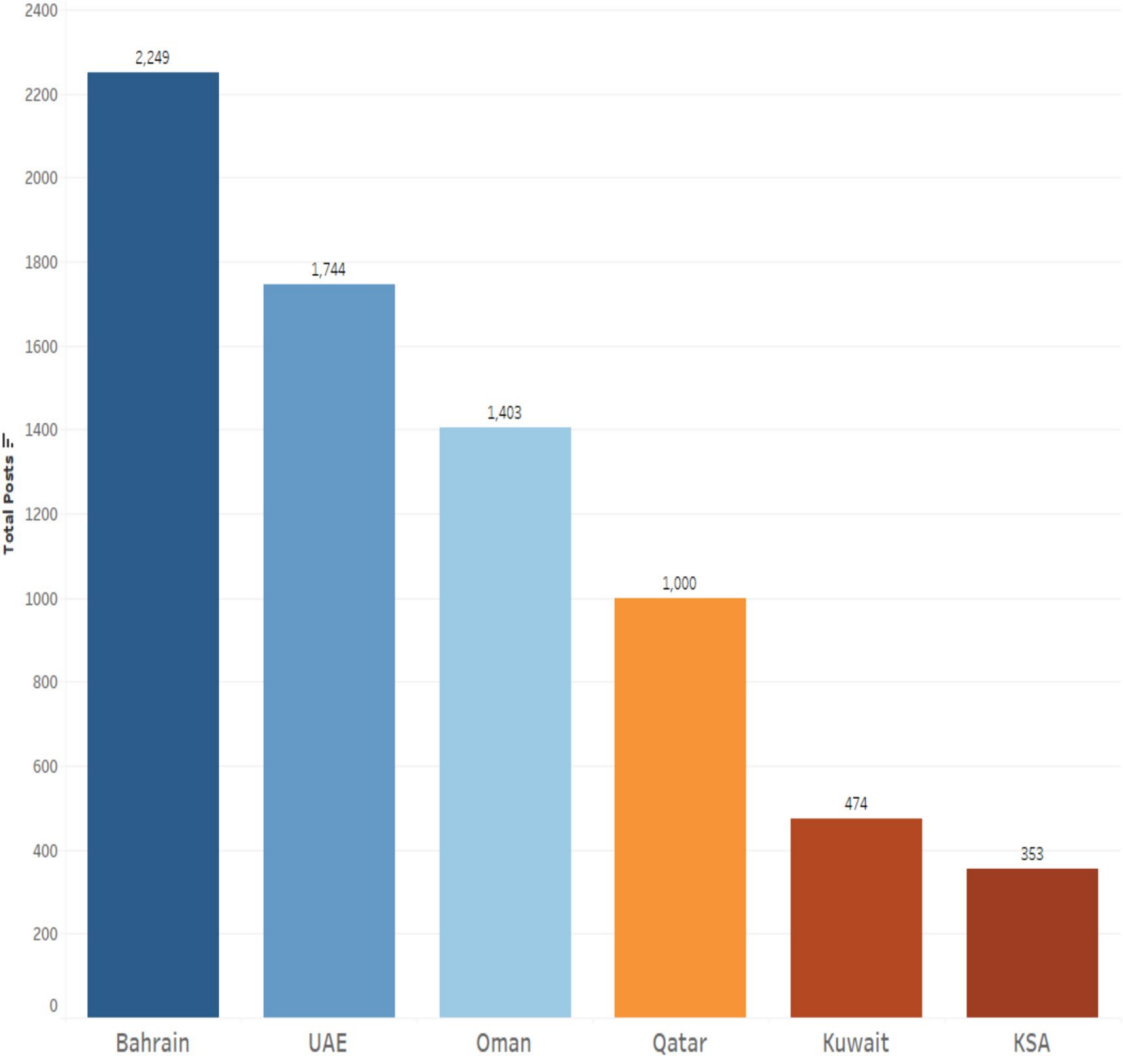
Number of Instagram Posts (1st May 2021 to 1st April 2022) across GCC Countries

The GCC ministries of health utilized Instagram to demonstrate a consistent and strategic approach to communicating essential COVID-19-related information. Our first research question (RQ1) provides insight into how GCC ministries of health (MOH) utilized Instagram in managing COVID-19 messages. In general, the health ministries from Bahrain, the UAE, and Oman were the most active on Instagram. As depicted in Fig. 4, Bahrain had 2,249 posts on Instagram during the COVID-19 pandemic, indicating a high level of posting activity. Saudi Arabia had the lowest number of posts on its Instagram page. All posts analyzed from the health ministries of the six countries were courteous, respectful, and reassuring.

Our second question (RQ2) inquired about the nature of the content that ministries of health (MOHs) in the GCC nations disseminate on Instagram, and the extent to which the messaging conformed to the principles of the Crisis and Emergency Risk Communication (CERC) framework. The CERC framework highlights the significance of these themes as crucial elements, focusing on the provision of information and the promotion of activities to properly handle a crisis. Table 4 indicates that Instagram accounts from other countries also share content related to the CERC principles to different extents.

**Table 4:** Posts’ Themes Distribution among the Gulf Instagram accounts

Themes		Oman	KSA	Bahrain	Kuwait	Qatar	UAE
		**No. (%)**	**No. (%)**	**No. (%)**	**No. (%)**	**No. (%)**	**No. (%)**
**Risk & Crisis Information**	Background Information	0 (0%)	3 (2%)	3 (2%)	0 (100%)	4 (3%)	0 (0%)
	Symptoms/ Treatment	2 (1%)	18 (12%)	7 (5%)	0 (100%)	13 (9%)	10 (7%)
	Risk factors	0 (0%)	0 (0%)	0 (0%)	0 (100%)	0 (0%)	20 (13%)
	Vaccination, Infection & Death cases	74 (49%)	48 (32%)	12 (8%)	150 (100%)	13 (9%)	103 (69%)
	Inquires & Clarifications	7 (5%)	34 (23%)	23 (15%)	0 (100%)	32 (21%)	0 (0%)
	Lifting/ changing Restrictions	0 (0%)	1 (1%)	3 (2%)	0 (100%)	27 (18%)	0 (0%)
**Promoting Actions**		55 (37%)	33 (22%)	26 (17%)	0 (100%)	29 (19%)	13 (9%)
**Activities, Events & Campaigns**		8 (6%)	9 (6%)	45 (30%)	0 (100%)	21 (18%)	3 (2%)
**Reassurance**		3 (2%)	0 (0%)	8 (5%)	0 (100%)	1 (1%)	0 (0%)
**Collective Responsibility**		0 (0%)	3 (2%)	7 (5%)	0 (100%)	1 (1%)	0 (0%)
**General**		1 (1%)	1 (1%)	16 (11%)	0 (100%)	2 (1%)	1 (1%)
Total		150 (100%)	150 (100%)	150 (100%)	150 (100%)	150 (100%)	150 (100%)

As depicted in Table 4, all of Kuwait's Instagram posts align with aspects of the CERC standards, notably in the areas of "Background Information" and “Vaccination, Infection & Death cases,” since they allocate 100% of their posts to these categories. However, for Kuwait there are no posts in "Promoting Actions" or "Reassurance", which are also important aspects of the CERC principles.

Oman has a significant percentage of posts in “Promoting Actions” (37%), but lacks representation in key message areas. Kingdom of Saudi Arabia (KSA) and Bahrain show a more balanced distribution across the categories that are associated with CERC principles, such as "COVID-19 key messages" and “Promoting Actions.”

Our third research question (RQ3) inquired about how online audience engagement manifests itself on Instagram content in terms of likes and comments for the MOHs in the GCC nations. In this context, the online engagement of the audience with Instagram content, active engagement metrics of likes and comments [57], are clear markers of how the public perceives and responds to the health-related content shared by these official organizations. On Instagram, a heart symbol for likes denotes a user’s support for or endorsement of content. They provide people with a convenient and direct method to express their appreciation or agreement with the messages being shared. An abundance of likes on a post implies that the material strongly connects with the audience, demonstrating its significance and the successful conveyance of crucial health information.

Comments on Instagram posts enable the audience to articulate their opinions, inquire, and share personal views and anecdotes regarding the posted content. Comments can provide significant insights into the public's concerns, misconceptions, and information requirements surrounding health matters. In addition, they enable a two-way communication channel via which MOHs may directly interact with their audience, promoting a sense of community and confidence.

Likes and comments not only act as indicators of interaction but also enhance the overall visibility of the postings. The algorithm used by Instagram prioritizes material that has a higher level of user interaction, which in turn increases the chances of it being seen by a larger number of people [58]. This enhances the dissemination of vital health messages, potentially impacting public behavior and attitudes towards health warnings, vaccination programs, and other public health initiatives.

Therefore, understanding and analyzing these forms of online audience engagement can provide GCC nations' MOHs with important feedback on their communication strategies. It allows companies to customize their material to better align with the audience's requirements and interests, therefore improving the efficacy of their public health efforts on social media platforms such as Instagram. Research reveals that photos and faces garner a higher level of engagement in terms of likes and comments on Instagram [59].

**Fig. 5:**
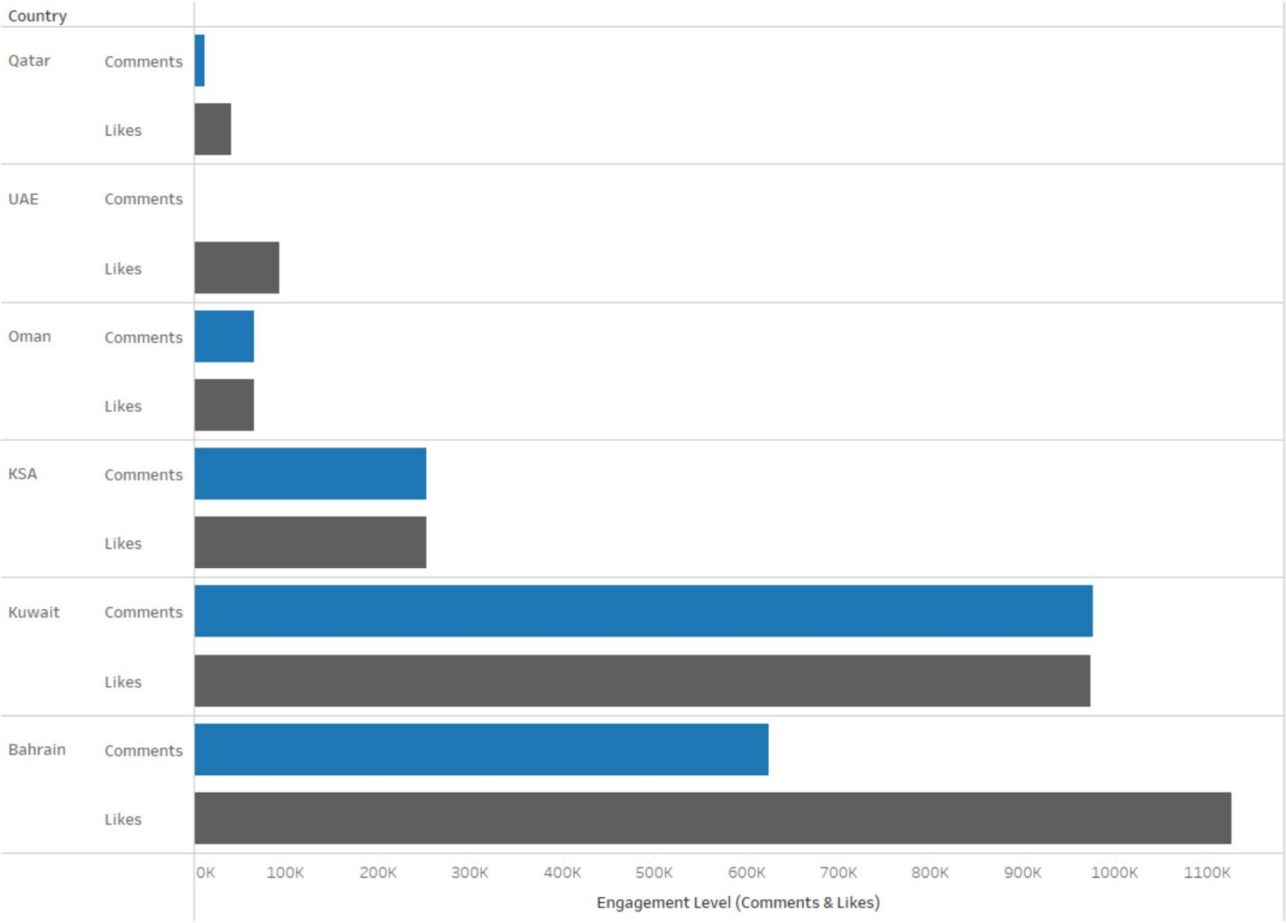
Total distribution of likes & comments among GCC countries

Figure 5 below demonstrates that Bahrain once more has the most interactions, but Kuwait is now in second place, having the largest share of total interactions.

Kuwait’s most popular post celebrated a day with zero COVID-19 deaths, amassing over 15,000 likes. In KSA, a post advocating the cessation of handshakes attracted 13,000 likes and upwards of 60 comments. Bahrain's daily infection update became one of the most engaged, with over 10,000 interactions, whereas the UAE's post emphasizing collective responsibility gained over 2,000 likes. The post from Oman's Instagram account, detailing current government activities, garnered the most significant engagement in terms of likes and comments. Qatar saw the highest interaction on posts about ongoing government actions, notably the introduction of new features in the Ehteraz COVID-19 app.

## Discussion

### MOHs instagram usage for Covid-19 related messages

During the COVID-19 pandemic, the strategic dissemination of vital health information has emerged as a crucial aspect of public health management [9]. The Gulf Cooperation Council (GCC) Ministries of Health (MOHs) have effectively leveraged Instagram for this purpose, showcasing a deliberate and informed approach to communicating essential COVID-19-related information. Our analysis, particularly focusing on the period from 1st May 2021 to 1st April 2022, reveals that Bahrain, the United Arab Emirates (UAE), and Oman were notably active on this platform. Bahrain led with an impressive tally of 2,249 posts, indicating a high level of engagement and commitment to utilizing social media as a tool for public health communication. In contrast, Saudi Arabia displayed the least activity, with the fewest posts among the GCC countries. This period saw the Ministries not only disseminating key COVID-19 messages but also promoting actions, highlighting activities and campaigns, offering reassurance, and fostering a sense of collective responsibility. The analysis further indicates that infographics and non-human subject images were particularly effective, achieving the highest levels of engagement and thereby underscoring the significant impact of content type on enhancing public interaction and response during health crises.

### GCC instagram use and CERC framework

In our investigation of the Gulf Cooperation Council (GCC) Ministries of Health’s utilization of Instagram for public health communication during the COVID-19 pandemic, we integrated the key principles of the Crisis and Emergency Risk Communication (CERC) framework. The CERC framework underscores the importance of disseminating key information and promoting actions critical for effective crisis management [13, 52]. Our findings, as detailed in Table 4 above, reveal a diverse adherence to CERC principles across the GCC, with a notable variation in content themes among the different countries.

However, Kuwait stands out for its different approach. It dedicates 100% of its Instagram posts to “Background Information” and “Vaccination, Infection & Death cases,” demonstrating a focused commitment to informing the public about the pandemic's fundamentals and the critical importance of vaccination. This singular focus, however, results in a noticeable absence of content in other crucial CERC categories such as “Promoting Actions” and “Reassurance,” which are essential for engaging and guiding the public through the crisis [7].

Conversely, Oman places a strong emphasis on “Promoting Actions,” with 37% of its posts dedicated to this category. This showcases a proactive approach to encouraging public health behaviors and actions. Nevertheless, Oman's content is lacking in key informational areas, which may compromise the comprehensiveness of its crisis communication strategy.

The approaches by KSA and Bahrain exhibit a more balanced distribution of content across CERC-related themes. Their approach reflects an integration of “COVID-19 key messages” with actions and responses, suggesting a strategic alignment with the CERC principles. This balanced approach likely facilitates a more rounded public understanding and engagement with the health crisis.

Our findings underscore the importance of adopting a diversified content strategy that embodies all facets of the CERC framework. While focusing on specific themes, such as vaccination, is crucial, our findings suggest that incorporating a wide range of content types—including key messages, action prompts, and reassurance—can enhance public engagement and adherence to health directives. This comprehensive approach is vital for effectively managing public health responses during crises [7].

Our research aligns with the findings of other studies, which indicate that all states within the Gulf Cooperation Council (GCC) successfully handled the pandemic. This was accomplished by improving their healthcare and public health infrastructures and accelerating changes, which under typical circumstances, may have advanced more slowly [60]. These governments are currently using the knowledge and insights gained from the initial stages of the pandemic to shape their policies and objectives. This is especially pertinent in the context of vaccination distribution and bolstering readiness for any prospective health crises. The proficient utilization of new technology across several sectors played a crucial role in effectively combating the virus in GCC nations, leading to successful mitigation of the pandemic [61].

### Online audience engagement

#### Dominant themes and enhanced public engagement

The cornerstone of exceptional crisis communication is effectively tailoring and broadcasting messages that deeply resonate with the public’s concerns and informational cravings, particularly amidst health emergencies like COVID-19. The analysis of this study pinpoints three pivotal themes—COVID-19 Key Messages, Prompting Actions, and Activities, Events, and Campaigns—prevalent in the GCC Ministries of Health’s posts. These themes demonstrate a high alignment with the (CERC) framework. A nuanced examination reveals diverse levels of public engagement across these themes, underscoring the vital influence of content specificity in fostering an engaged and well-informed public dialogue. For instance, Kuwait’s notable post commemorating a day without COVID-19 fatalities led the engagement with a staggering over 15,000 likes, while KSA’s innovative post advocating for the cessation of handshakes followed closely with 13,000 likes and over 60 comments. These figures not only illustrate the themes of Prompting Actions but also underscore the profound impact of message alignment with public sentiments in driving significant engagement.

#### The impact of infographic posts

Social media images engender a greater level of engagement [59, 62]. This investigation further solidifies the significant role of infographics in mobilizing public engagement on social platforms, affirming the elevated interaction levels associated with visually represented information. The study revealed that Bahrain’s daily infection updates, through adept use of infographics, captivated the audience with over 10,000 interactions, showcasing the high efficacy of visual content. Similarly, the UAE’s post advocating collective responsibility through a compelling infographic presentation garnered over 2,000 likes, reinforcing the crucial role of visually appealing content in disseminating complex health messages succinctly. These statistics not only highlight the preferential engagement with infographic posts but also accentuate their pivotal role in magnifying the effectiveness of crisis communication strategies by making intricate health advisories more accessible and engaging to the general public.

On the other hand, delving deeper into the content analysis and its correlation with public engagement levels, an interesting trend emerges: posts featuring non-human and non-gender specific imagery tend to resonate more with audiences, as evidenced by the high levels of engagement with such posts across the GCC. This phenomenon was notably observed in Oman and Qatar’s most engaging posts, which detailed current government actions and introduced new features in the Ehteraz COVID-19 app, respectively, without focusing on human elements. This trend not only suggests that the absence of human imagery does not hinder, but may indeed amplify public engagement but also signals a broader strategic insight for crisis communication. Research has shown that infographics receive greater levels of engagement on Instagram in the form of likes and comments [7]. It highlights the untapped potential of non-human imagery to evoke strong public responses, thereby offering a novel approach to enhancing engagement with health communication strategies.

During our analysis of Instagram usage by the GCC Ministries of Health, a few anomalies were identified that merited special attention. Anomalies, in this context, refer to data points or patterns in communication that deviated significantly from the general trends observed across the region. For example, the extraordinarily high engagement rates on certain posts, such as Kuwait’s commemoration of a day without COVID-19 fatalities, initially appeared as outliers. These anomalies were rigorously examined to determine their causes and implications for our broader findings.

To handle these anomalies, we first verified the authenticity and accuracy of the data to rule out any errors in data collection or processing. Subsequently, we conducted a deeper analysis to understand the context surrounding these high-engagement posts, considering factors such as timing, content, and external events. This approach helped us ascertain whether these were genuine instances of effective communication or if they were influenced by external variables such as national events or global news impacting public sentiment.

### Relevant health policies

Throughout the pandemic, health policies within the GCC have been dynamic, evolving from immediate containment measures to long-term strategies focusing on vaccination and public safety. The MOHs’ strategic use of Instagram content reflects this evolution, shifting from early emphasis on quarantine measures to robust promotion of vaccination drives as the pandemic progressed. This adaptive communication strategy was crucial in keeping the public informed and engaged, facilitating a smoother implementation of evolving health policies.

Moreover, an analysis of engagement metrics reveals that posts directly supporting health policies, like Bahrain’s frequent updates on infection rates and vaccination progress, not only achieved high engagement rates but also likely influenced public behavior in positive ways. Such data underscore the efficacy of aligning communication strategies closely with policy objectives. In another instance, Kuwait’s intensive Instagram campaign to encourage vaccination mirrors its national health objective to achieve widespread vaccine uptake, which is critical in managing public health during a pandemic. However, gaps were noted, such as in Oman, where a narrower focus on information diversity may have limited the effectiveness of these communication strategies. To enhance future crisis communication, it is recommended that MOHs ensure a balanced content approach that covers a wide array of informational needs—from prevention tips to updates on policy changes—thereby supporting a comprehensive public health strategy.

These insights not only underscore the potential of social media as a powerful tool for health communication but also suggest a model for integrating digital communication tools with health policy implementation. Future strategies should prioritize real-time updates and proactive public engagement during policy announcements to improve public compliance and participation. Engaging with the public through interactive formats like Q&A sessions and live updates can further enhance transparency and trust, establishing a more responsive health communication framework for future crises.

## Conclusion

Overall, examining the use of Instagram for public health messages in the GCC during the COVID-19 epidemic provides useful insights into the platform's efficacy as a communication tool during times of crisis. It aids in identifying optimal methods, obstacles, and strategic paths for using digital platforms to bolster public health goals.

Future strategies should prioritize augmenting interactive engagement on Instagram by promoting user involvement through Q&A sessions, live debates, and interactive narrative elements. Timely and enlightening replies to comments have the potential to establish confidence and reliability. Collaborating with well-known influencers can assist public health authorities in the GCC region to expand their influence, particularly among younger demographics, thereby making their messages more relatable. Adapting information to incorporate the varied cultural subtleties of the GCC can result in improved compliance with public health guidelines. These insights should inform the creation of strategic communication plans, integrating the knowledge gained from the COVID-19 epidemic into complete frameworks for crisis communication. This preparedness will facilitate the expedient and efficient utilization of social media in addressing forthcoming health crises.

## Data Availability

The supporting documents and data for this study can be made available and shared when requested.

## Appendix

**Table:** The codebook used in this study

#	Theme	Sub-Theme	Message Content	2nd Dimension:	3rd Dimension:
**1**	**COVID-19 key Messages** (Lundgren & Andrea, 2018; [51] Mohamed, 2021; [55] Malik et al., 2021) [7]	Background Information	Dissemination of basic information on the nature of the virus and the way it evolves	1- Image Type: (Infographic/ Photography) 2- Human-subject Image: (Caregiver/ Celebrity/ ordinary individual/ none) 3- Gender Depiction: (Male/ Female/ both/ none)	Number of Likes: (0–1000/ 1001–5000/ Over 5000
		Symptoms & Treatment	Describe the symptoms of the disease or update any information related to the treatment method		
		Risk outbreak factors	Draw the factors that cause the transmission of the virus on a large scale		
		Infection & Death cases	View statistics of infected cases and death numbers		
		Inquires & Clarifications	Clarifying concepts and answering common questions		
		Lifting Restrictions	Announcing the lifting of the ban or the easing of distancing restrictions		
**2**	**Promoting Actions** (Lundgren & Andrea,2018; [51] Mohamed, 2021 [55]; Malik et al., 2021) [7]	Prompting actions	Encourage people to do certain actions to stay safe		
**3**	**Activities, Events & Campaigns** (Lundgren & Andrea, 2018; [51^51^] Mohamed, 2021; [55] Malik et al., 2021) [7]	Current activities	An explanation of the government's current activities regarding the pandemic		
		Upcoming events	An explanation of the government's upcoming activities regarding the pandemic		
**4**	**Reassurance** (Lundgren& Andrea,2018; [51] Mohamed, 2021; [55] Malik et al., 2021) [7]	Showing gratitude & expressing truthfulness	Acknowledging people's feelings and showing empathy		
**5**	**Collective Responsibility** (Lundgren& Andrea,2018; [51] Mohamed, 2021; [55] Malik et al., 2021) [7]	Social & common responsibilities	Encouraging people to cooperate and engage in responsibility to limit the spread of the pandemic		
6	**General**	-	Posts that do not follow any of the above themes		

## Abbreviations

CERC: Crisis and Emergency Risk Communication
GCC: The Gulf Cooperation Council
MOHs: Ministries of Health
